# Seminal Plasma Enhances Cervical Adenocarcinoma Cell Proliferation and Tumour Growth In Vivo

**DOI:** 10.1371/journal.pone.0033848

**Published:** 2012-03-19

**Authors:** Jason R. Sutherland, Kurt J. Sales, Henry N. Jabbour, Arieh A. Katz

**Affiliations:** 1 MRC/UCT Research Group For Receptor Biology, Institute of Infectious Disease and Molecular Medicine and Division of Medical Biochemistry, Faculty of Health Sciences, University of Cape Town, Cape Town, South Africa; 2 MRC Human Reproductive Sciences Unit, The Queen's Medical Research Institute, The University of Edinburgh, Edinburgh, United Kingdom; University of Nebraska Medical Center, United States of America

## Abstract

Cervical cancer is one of the leading causes of cancer-related death in women in sub-Saharan Africa. Extensive evidence has shown that cervical cancer and its precursor lesions are caused by Human papillomavirus (HPV) infection. Although the vast majority of HPV infections are naturally resolved, failure to eradicate infected cells has been shown to promote viral persistence and tumorigenesis. Furthermore, following neoplastic transformation, exposure of cervical epithelial cells to inflammatory mediators either directly or via the systemic circulation may enhance progression of the disease. It is well recognised that seminal plasma contains an abundance of inflammatory mediators, which are identified as regulators of tumour growth. Here we investigated the role of seminal plasma in regulating neoplastic cervical epithelial cell growth and tumorigenesis. Using HeLa cervical adenocarcinoma cells, we found that seminal plasma (SP) induced the expression of the inflammatory enzymes, prostaglandin endoperoxide synthase (PTGS1 and PTGS2), cytokines interleukin (IL) -6, and -11 and vascular endothelial growth factor-A(VEGF-A). To investigate the role of SP on tumour cell growth in vivo, we xenografted HeLa cells subcutaneously into the dorsal flank of nude mice. Intra-peritoneal administration of SP rapidly and significantly enhanced the tumour growth rate and size of HeLa cell xenografts in nude mice. As observed in vitro, we found that SP induced expression of inflammatory PTGS enzymes, cytokines and VEGF-A in vivo. Furthermore we found that SP enhances blood vessel size in HeLa cell xenografts. Finally we show that SP-induced cytokine production, VEGF-A expression and cell proliferation are mediated via the induction of the inflammatory PTGS pathway.

## Introduction

At present, there are more than 273,000 deaths worldwide from cervical cancer each year accounting for 9% of total cancer deaths in women [1]. In sub-Saharan Africa, cervical cancer is one of the leading causes of cancer related death in women [2]. Infection with high risk HPV is the most common risk factor for the development of cervical cancer, with HPV 16 accounting for at least 50% of malignancies [3]. Furthermore, other factors including a weakened immune system and sexually transmitted infection by Chlamydia trachomatis and Neisseria gonorrhoeae can contribute to the etiology of cervical cancer [4].

Persistent virus infection, such as that caused by HPV has been associated with chronic inflammation and cancer [5]. A link between high risk HPV infection of cervical epithelial cells and the prostaglandin endoperoxide synthase (PTGS also called cyclooxygenase; COX) pathway has been established [6]. These findings suggest that HPV-mediated tumour growth may be regulated by inflammatory pathways. However the detailed cellular and molecular mechanisms and inflammatory pathways responsible for cervical cancer progression following HPV infection remain to be fully determined.

Although simplistically regarded as an alteration in immune cell recruitment to sites of injury and repair, inflammation involves alterations to epithelial, vascular and immune cell function. These are orchestrated by specific molecular pathways involving a host of growth factors, cytokines, chemokines and lipid mediators, which initiate tissue regeneration and repair mechanisms and alters the function of the vasculature and the endothelium to enhance angiogenesis, vascular permeability and the extravasation of immune cells from the blood to the inflamed tissue [7]. The link between chronic inflammation and cancer has been long established [8], [9]. Our laboratory and others have demonstrated elevated expression of PTGS1, PTGS2, the E-series prostanoid receptors PTGER2 and PTGER4 together with enhanced biosynthesis and signaling of PGE_2_ in cervical carcinomas [10], [11], [12], [13]. These findings suggest an important regulatory mechanism for this inflammatory axis in cervical cancers. This pro-inflammatory pathway can be induced by a variety of stimuli, including lipopolysaccharides (LPS), cytokines, growth factors and tumour-promoting chemical carcinogens [14]. Moreover in sexually active women, in the absence of barrier contraceptive, the inflammatory environment can be further modulated by mediators present in seminal fluid [15], [16], [17]. Seminal fluid is comprised of a vast diversity of antigenically distinct molecules that include cytokines, angiogenic factors, prostaglandins, proteases, protein kinases, transporter proteins, structural molecules and immune response proteins [18], [19]. In several species, seminal fluid has been shown to interact with the epithelial lining of the reproductive tract and activate the innate and adaptive immune system [20], [21]. This promotes the influx of immune cells such as dendritic cells, granulocytes and leukocytes and activates and expands inducible regulatory T cell populations to promote immune tolerance and prepare the reproductive tract for conception [22], [23], [24]. However, little is known of the effect of seminal plasma (SP) on the neoplastic cervical epithelium of sexually active women. SP can promote the release of matrix metalloproteinases (MMPs) which can enhance metastases [25], [26], as well as promote the release of local pro-inflammatory mediators such as IL-1ß and IL-8 to regulate the tumour microenvironment. Furthermore, components of SP can enter into the endometrial bed or the peritoneal bed as a result of hematogenous dissemination or direct tissue perfusion through the anterior or posterior vaginal fornix after sexual intercourse [27]. It is therefore plausible that in addition to the direct regulation of cell surface proteins by SP, repeated exposure of the cervico-vaginal interface to seminal fluid could result in an alteration in the local levels of inflammatory mediators which could enhance cervical tumorigenesis.

Here we investigated the impact of seminal plasma on neoplastic cervical epithelial cell growth and tumorigenesis. Using HeLa cervical adenocarcinoma cells, we found that seminal plasma (SP) induced the expression of the inflammatory enzymes, prostaglandin endoperoxide synthase (PTGS1 and PTGS2), cytokines IL-6, IL-11 and the angiogenic factor VEGF-A in vitro. We subsequently investigated the role of SP in inflammation-induced tumorigenesis in vivo by xenografting HeLa cells subcutaneously in the dorsal flank of nude mice. Following engraftment, mice were injected with SP by intraperitoneal injection three times weekly. Administration of SP rapidly and significantly enhanced the tumour growth rate and size of HeLa cell xenografts. As observed in vitro, we found that SP induced expression of PTGS1, PTGS2 and the PG receptors PTGER1, PTGER2 and PTGER4 as well as inflammatory cytokines IL-6 and IL-11 in vivo. Furthermore we found that SP enhanced the expression of VEGF-A and blood vessel size in HeLa cell xenografts. Finally we show a mechanism for the SP-induced IL-6, IL-11, VEGF-A and HeLa cell proliferation in vitro via the induction of the inflammatory PTGS pathway.

## Methods

### Ethics statement

#### Human Ethics

Ethics approval for the study was obtained from the University of Cape Town Research Ethics Committee (REC/REF: 152/99). Written informed consent was obtained from all subjects before sample collection.

#### Animal Ethics

All mouse experiments were performed according to protocols approved by the University of Cape Town Animal Research Ethics Committee (approval no. REC/REF:006/044).

### Reagents

Culture medium, penicillin-streptomycin and fetal-calf serum was purchased from Highveld Biological (PTY) Limited (Cape Town, South Africa). PBS, BSA, Percoll and Tri-reagent® were purchased from Sigma Chemical Company (Cape Town, South Africa). CellTitre96 Aqueous One Solution Proliferation Reagent was purchased from Promega Corp. (Madison, WI, USA). Ki-67 and CD31 antibodies were purchased from Vector Laboratories (Peterborough, UK) and Abcam (Cambridge, UK) respectively.

### Cell culture

Wild type HeLa-S3 cells were purchased from BioWhittaker (Berkshire, UK) and were maintained in DMEM nutrient mixture F-12 with Glutamax-1 and pyridoxine supplemented with 10% fetal bovine serum and 1% penicillin-streptomycin (500 IU/ml penicillin and 500 µg/ml streptomycin) at 37°C and 5% CO_2_ (v/v).

### Semen donors and preparation

Semen was obtained from 10 healthy males attending the Andrology Laboratory of the Reproductive Medicine Unit at Groote Schuur Hospital, Cape Town. Ejaculates were collected in sterile specimen jars following voluntary self-masturbation. Days of sexual abstinence prior to ejaculation were self-reported and sample volume, sperm count, pH and viscosity were noted. All samples were processed within 2–4 hours after collection. The ten individual ejaculates were transported to the laboratory and were pooled and overlayed on a 100-50% percoll gradient. Seminal plasma (SP) was isolated from the pooled ejaculate by density gradient centrifugation at 500 *g* for 20 min. The seminal plasma was aliquoted and stored at −80°C until required. Prior to injection, the seminal plasma was thawed on ice and diluted in sterile filtered serum free medium to use at a concentration of 1∶100 for in vitro studies and diluted in sterile filtered PBS to a dilution of 1∶500 for in vivo studies. Seminal fluid has been reported to exert no effect on HeLa cell viability up to and including a dilution of 1∶50 [25].

### Xenograft experiments

A suspension of 2×10^6^ HeLa cells in a total volume of 0.2 ml PBS was injected subcutaneously into the right dorsal flank of MF-1 nude mice. Following engraftment, the mice were assigned into two treatment groups, Control (n = 8) and SP (n = 6). The mice were injected three times weekly with 100 µl PBS or SP (1∶500) via intra-peritoneal injection for ten weeks. At the end of the study animals were sacrificed and tumours excised. A portion of the tumour was fixed in 0.2% paraformaldehyde for wax-embedding and immunohistochemistry and the remainder used for RNA extraction. The animals were maintained under sterile conditions in individually vented cages.

### Immunohistochemical analysis

Immunohistochemistry was performed on nude mouse xenografts (n = 5 control, n = 5 SP treated animals) using standard techniques as previously described [28]. Following antigen retrieval, sections were blocked in 5% normal goat serum (Ki-67) or normal porcine serum (CD31) diluted in Tris-buffered saline (TBS) with 5% BSA. Tissue sections were incubated with mouse anti-human Ki-67 polyclonal antibody (1∶200) or rabbit anti mouse/human CD31 polyclonal antibody (1∶250) overnight at 4°C. Control tissue was incubated with immunoglobulin (IgG) from the host species. Subsequently sections were incubated with goat anti-mouse biotinylated or porcine anti-rabbit biotinylated antibodies (DAKO, Cambridgeshire, UK), followed by streptavidin-horse radish peroxidase complex (DAKO). Colour reaction was developed with 3′3 diaminobenzidine (DAKO), and sections were counterstained in haemotoxylin. The number of positively stained cells for Ki-67 was quantified using standard stereological techniques. Briefly, each section was examined using a ×40 objective from a Leitz DMRB microscope (Leica Microsystems Wetzlar, Germany) fitted with an automatic stage (Prior Scientific Instruments Ltd, Cambridge, UK) using a live-feed camera (JVC-KYF55B; Yokohama, Japan) and were analyzed using Image-Pro Plus 6.2 software (Media Cybernetics, Wokingham, Berkshire, UK). A total of 20 randomized fields of view were examined and counted from a total of five individual control and five individual SP xenograft tumours. Data are expressed as mean number of positively stained cells per tumour examined. Blood vessel size in the xenografts (n = 5 Control, n = 5 SP treated animals) was measured in sections that were stained with CD31 to highlight the blood vessel perimeter. A total of 100 randomized fields of view were examined for control and treated animal. Blood vessel diameter was measured using Image-Pro Plus 6.2 software. Data are represented as mean size of blood vessels per µm^2^ of tumour examined.

### Taqman quantitative RT-PCR

HeLa cell and xenograft RNA was extracted using Tri-reagent (Qiagen, Crawley, UK) following the manufacture's guidelines. RNA samples were reverse transcribed using VILO (Invitrogen, Paisley, UK). Quantitative RT-PCR was performed under standard operating conditions using an ABI Prism 7500 (Applied Biosystems, Warrington, UK), using sequence-specific primers and 6-carboxyfluoresceine-labelled probes as detailed in table 1. Expression of PTGS1, PTGS2, PTGER receptors, IL-6, IL-11, VEGF-A and Ki-67 were normalized to RNA loading for each sample using the 18S ribosomal RNA as an internal standard. Data were analysed and processed using Sequence Detector v1.6.3 (Applied Biosystems). Results are expressed relative to an endogenous control of HeLa cell cDNA included in each experiment or expressed as a fold increase determined by dividing the relative expression of the treatment group by the relative expression of the control group. Data are presented as mean ± SEM.

**Table 1:** Sequences of Taqman primers and probes for Real Time (RT-PCR).

Target Gene	Sequence of Primers and Probe (5′-3′)
PTGS-1 Forward primer	TGTTCGGTGTCCAGTTCCAATA
PTGS-1 Reverse primer	ACCTTGAAGGAGTCAGGCATGAG
PTGS-1 Probe (FAM)	CGCAACCGCATTGCCATGGAGT
PTGS-2 Forward primer	CCTTCCTCCTGTGCCTGATG
PTGS-2 Reverse primer	ACAATCTCATTTGAATCAGGAAGCT
PTGS-2 Probe (FAM)	TGCCCGACTCCCTTGGGTGTCA
PTGER1 Forward primer	AGATGGTGGGCCAGCTTGT
PTGER1 Reverse primer	GCCACCAACACCAGCATTG
PTGER1 Probe (FAM)	CAGCAGATGCACGACACCACCATG
PTGER2 Forward primer	GACCGCTTACCTGCAGCTGTAC
PTGER2 Reverse primer	TGAAGTTGCAGGCGAGCA
PTGER2 Probe (FAM)	CCACCCTGCTGCTGCTTCTCATTGTCT
PTGER3 Forward primer	GACGGCCATTCAGCTTATGG
PTGER3 Reverse primer	TTGAAGATCATTTTCAACATCATTATCA
PTGER3 Probe (FAM)	CTGTCGGTCTGCTGGTCTCCGCTC
PTGER4 Forward primer	ACGCCGCCTACTCCTACATG
PTGER4 Reverse primer	AGAGGACGGTGGCGAGAAT
PTGER4 Probe (FAM)	ACGCGGGCTTCAGCTCCTTCCT
IL-6 Forward primer	GCCGCCCCACACAGACA
IL-6 Reverse primer	CCGTCGAGGATGTACCGAAT
IL-6 Probe (FAM)	CCACTCACCTCTTCAGAACGAATTGACAAAC
IL-11 Forward primer	CCCAGTTACCCAAGCATCCA
IL-11 Reverse primer	AGACAGAGAACAGGGAATTAAATGTGT
IL-11 Probe (FAM)	CCCCAGCTCTCAGACAAATCGCCC
VEGF-A Forward primer	TACCTCCACCATGCCAAGTG
VEGF-A Reverse primer	TAGCTGCGCTGATAGACATCCA
VEGF-A Probe (FAM)	ACTTCGTGATGATTCTGCCCTCCTCCTT
Ki-67 Forward primer	GGACTTGCACGACTAA
Ki-67 Reverse primer	CCGTACGTCAATTGAC
Ki-67 Probe (FAM)	TTCGAACTGATCAT
18 s Forward primer	CGGCTACCACATCCAAGGAA
18 s Reverse primer	GCTGGAATTACCGCGGCT
18 s Probe (FAM)	TGCTGGCACCAGACTTGCCCTC

### Cell Proliferation

HeLa cells were seeded in 6 well plates at a density of 2×10^5^ cells per well in a final volume of 2 ml and allowed to propagate overnight before serum starvation for 24 hours. Thereafter cells were treated with SP (1∶100) or vehicle for 6 hours and the RNA subjected to real time RT-PCR analysis for Ki67 as described above. To investigate the expression of Ki-67 protein in HeLa cells, cells were seeded at a density of 5×10^4^ cells per well in a final volume of 1 ml in 2 well-chamber slides and allowed to adhere for 24 hrs before serum starvation overnight. The next day, cells were treated with SP (1∶500) or vehicle for 24, 48 or 72 hrs. Following treatment, cells were washed with PBS and fixed with 4% paraformaldehyde (diluted in PBS) for 10 min. Thereafter, the cells were subjected to two washes in TBS (50 mM Tris/HCl, 150 mM NaCl, pH 7.4) and blocked using 5% normal swine serum diluted in TBS for 30 min at room temperature. Cells were then incubated with the specific primary antibody for Ki-67 (1∶200) at 4°C overnight. The following day, the cells were incubated with 1∶250 dilution of swine-anti-rabbit biotinylated secondary antibody (DAKO, Cambridge, UK). The slides were incubated with streptavidin peroxidase (1∶1000) and detected with 3′3 diaminobenzidine (DAKO). Slides were counterstained with haemotoxylin and the number of positively stained cells for Ki-67 quantified by stereologer using Image-Pro Plus 6.2 software as described above. The effect of inhibition of PTGS enzymes on HeLa cell proliferation was determined using the CellTitre96 Aqueous One Solution Proliferation Reagent (Promega Corp) as per the manufacturer's protocol. HeLa cells were seeded in 96-well plates at a density of 3000 cells per well and allowed to propagate overnight before serum starvation for 24 hrs. Thereafter cells were treated with SP (1∶100) or vehicle for 24, 48 and 72 hrs. Data are presented as mean ± SEM of four independent experiments.

### Statistical Analysis

The data in this study was analyzed by t-test or one-way ANOVA using Graph Pad Prism 4.0c (Graph Pad, San Diego, CA). Where data are presented as fold increase, all statistical analysis was performed on untransformed data prior to conversion to fold increase.

## Results

### Upregulation of PTGS expression in HeLa cells in response to treatment with seminal plasma

**Figure 1:**
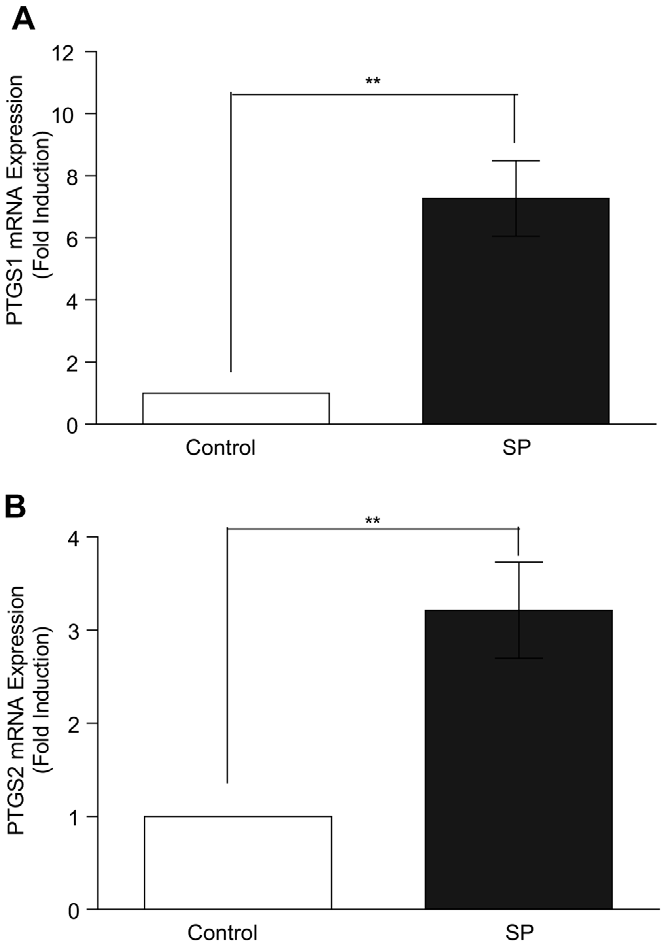
Upregulation of PTGS expression in HeLa cells in response to treatment with seminal plasma. (**A**) PTGS1 and (**B**) PTGS2 mRNA expression was measured by quantitative RT-PCR analysis and is significantly increased by SP (1∶100) as compared to vehicle (PBS) control, (both P<0.01**). Data are represented as mean ± SEM from 4 independent experiments.

We investigated whether exposure of neoplastic cervical epithelial cells to seminal plasma would induce expression of the inflammatory prostaglandin endoperoxide synthase (PTGS) pathway, which has been shown to promote tumorigenesis. HeLa cervical adenocarcinoma cells were treated with 1∶100 dilution of SP or vehicle (PBS) for 6 hours and the mRNA expression of PTGS1 and PTGS2 was determined by quantitative RT-PCR analysis. We found that SP significantly elevated expression of PTGS1 (Figure 1A; 7.3±2.7 fold increase compared to vehicle treatment; P<0.01) and PTGS2 (Figure 1B; 3.4±0.5-fold increase compared to vehicle treatment; P<0.01).

### Seminal plasma induces expression of pro-inflammatory cytokines IL-6, IL-11 and pro-angiogenic factor VEGF-A in HeLa cells

**Figure 2:**
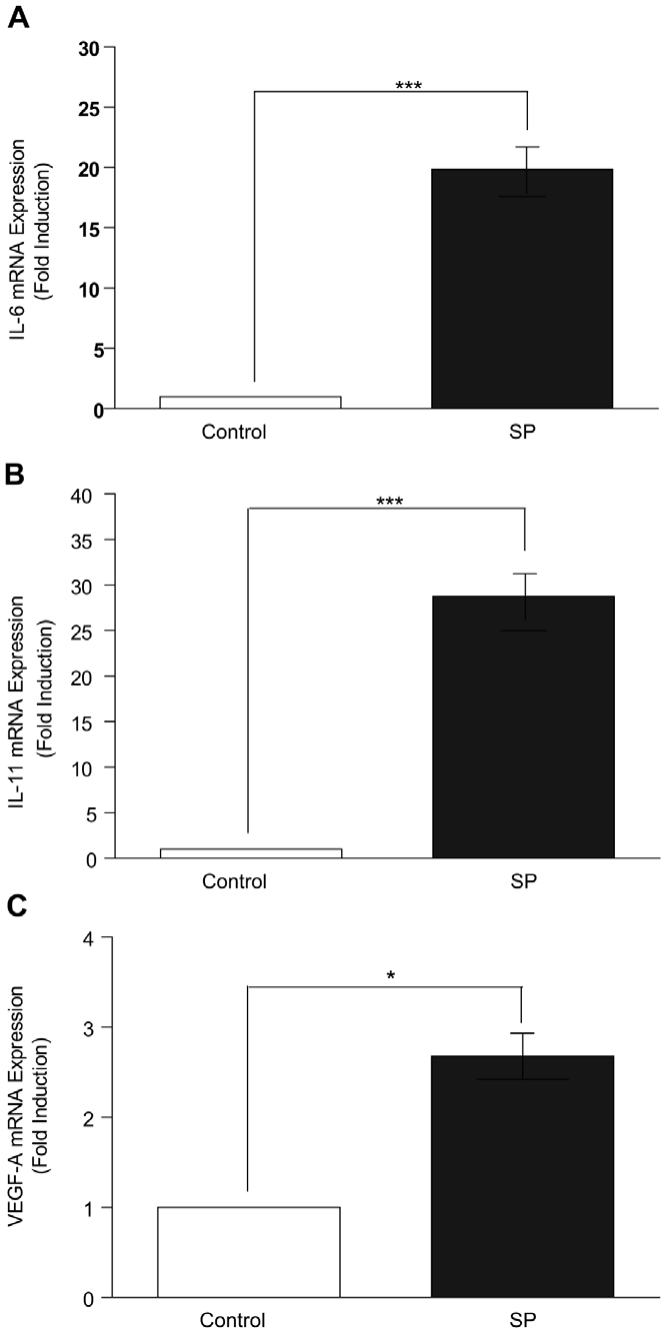
Increased expression of pro-inflammatory and pro-angiogenic mediators IL-6, IL-11 and VEGF-A in HeLa cells in response to treatment with seminal plasma. (**A**) IL-6, (**B**) IL-11 and (**C**) VEGF-A mRNA expression as determined by quantitative RT-PCR analysis are significantly increased in HeLa cells treated with SP (1∶100) in comparison to the vehicle (PBS) control treatment group, (*P<0.05, P<0.001***). Data are represented as mean ± SEM from 6 independent experiments.

Having established that SP induces the expression of PTGS1 and PTGS2, we next investigated the effect of SP on the expression of potent pro-inflammatory cytokines IL-6 and IL-11 and the pro-angiogenic factor VEGF-A by quantitative RT-PCR analysis. Treatment of HeLa cells with SP (1∶100) for 6 hrs significantly increased the expression of IL-6 (Figure 2A, 19.8±2.3 fold; P<0.001), IL-11 (Figure 2B, 28.8±3.8 fold; P<0.001) and VEGF-A (Figure 2C, 2.9±0.9 fold; P<0.05) mRNA compared to vehicle treatment.

### Seminal plasma enhances growth and weight of xenograft tumours in vivo

**Figure 3:**
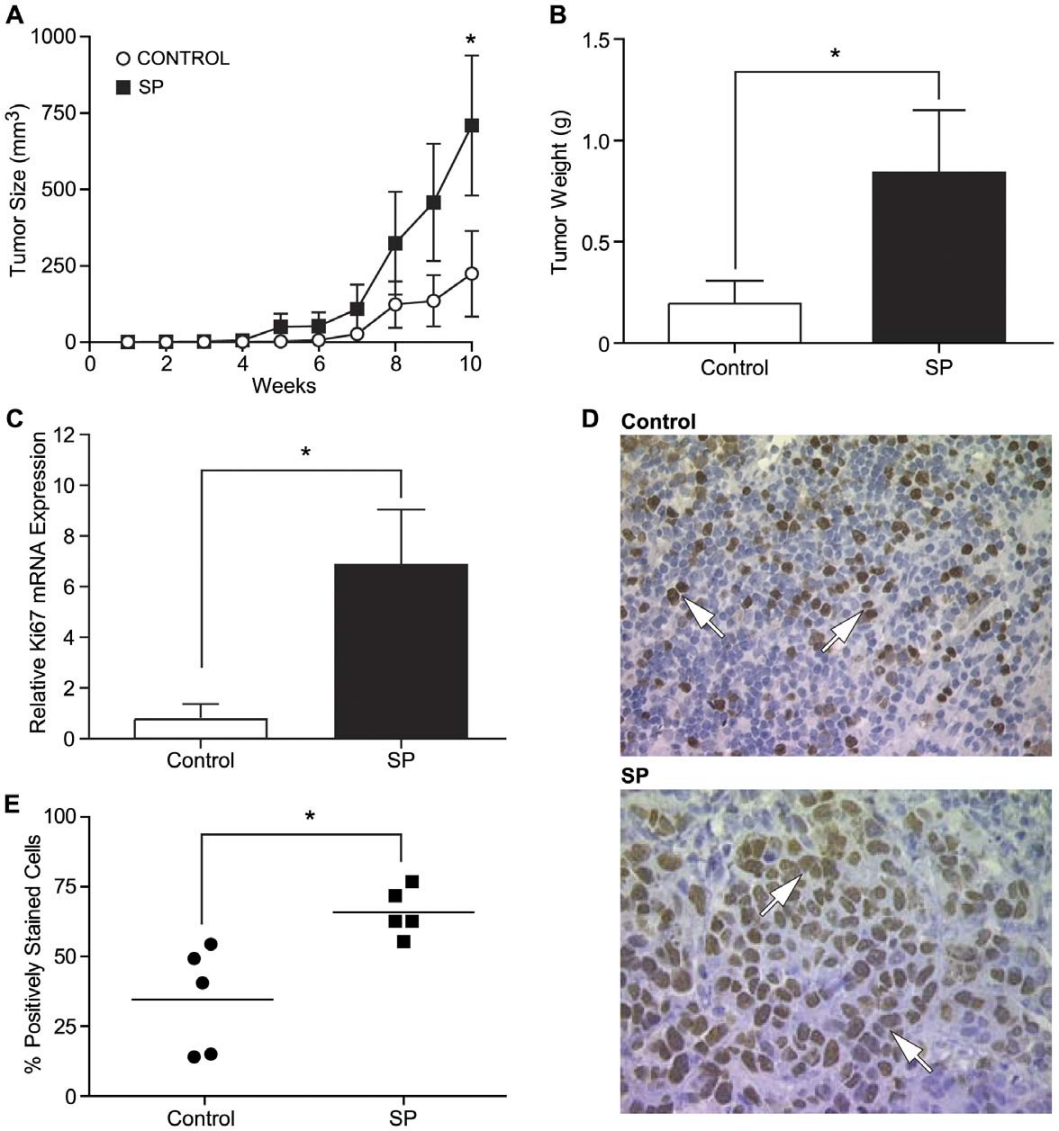
Seminal plasma enhances growth and weight of xenograft tumours in vivo. The effects of SP on tumour growth and tumour weight. MF-1 nude mice were engrafted with HeLa cells and treated with SP (n = 6) or vehicle (PBS) controls (n = 8). (**A**) Tumour size in the nude mice xenografts is significantly different after 10 weeks of SP injections in comparison to PBS treated mice (P<0.05*). (**B**) The weight of the tumours was determined at the end of the 10 week experimental period. Mice injected with SP displayed a significantly increased tumour weight in comparison to the control group (P<0.05*). (**C**) Ki-67 mRNA expression was measured by quantitative RT-PCR analysis and is significantly increased by SP (1∶500) as compared to the controls, (P<0.05*). (**D**) Immunohistochemical staining of Ki67 in SP-treated and control tumours from HeLa cell xenografts (n = 5 per group). (**E**) Number of positively stained cells for Ki67 was quantified and is significantly increased when treated with SP (1∶500) in comparison to the vehicle control treatment group (P<0.05*). Data are represented as mean ± SEM.

Components of SP, including prostaglandins, can enter into the endometrial bed or the peritoneal bed as a result of hematogenous dissemination or direct tissue perfusion through the anterior or posterior vaginal fornix after sexual intercourse [27]. We investigated whether SP could enhance tumorigenesis in vivo via the elevation of inflammatory mediators present systemically. We engrafted HeLa cells subcutaneously into the dorsal flank of MF-1 nude mice. Following engraftment, animals were administered SP (1∶500 dilution) or PBS (control group) three times a week via intra-peritoneal injection and tumour growth was measured over a 10 week period. We found a significant divergence in the growth of tumours in animals administered with SP compared with control, which was significant at 10 weeks (Figure 3A; P<0.05). Upon excising the tumours at the end of the 10 week treatment period, the tumours removed from the SP treated group also weighed significantly more than the control group confirming the SP-induced increase in tumorigenesis (Figure 3B; P<0.05). Having ascertained a significant increase in tumour size and weight in response to SP administration, we next confirmed that the enhanced tumour size and weight was due to induction of tumour cell proliferation by SP. Quantitative RT-PCR analysis for the proliferation marker Ki-67 revealed a 7.5±2.2 fold elevation in proliferation rate in xenograft tumours from SP treated mice compared with control mice (Figure 3C; P<0.05). This increase in proliferation index of xenograft tumours in mice treated with SP was further confirmed by immunohistochemistry with antisera for Ki-67 (indicated by the increased brown staining in SP treated tumours, arrowed in Figure 3D) and by quantitative stereology, which displayed 66±4 percent Ki-67 positive immunoreactivity in SP treated tumours in comparison to 35±9 percent in control tumours (Figure 3E; P<0.05).

### Upregulation of PTGS and PTGER expression in xenograft tumours in response to treatment with seminal plasma

**Figure 4:**
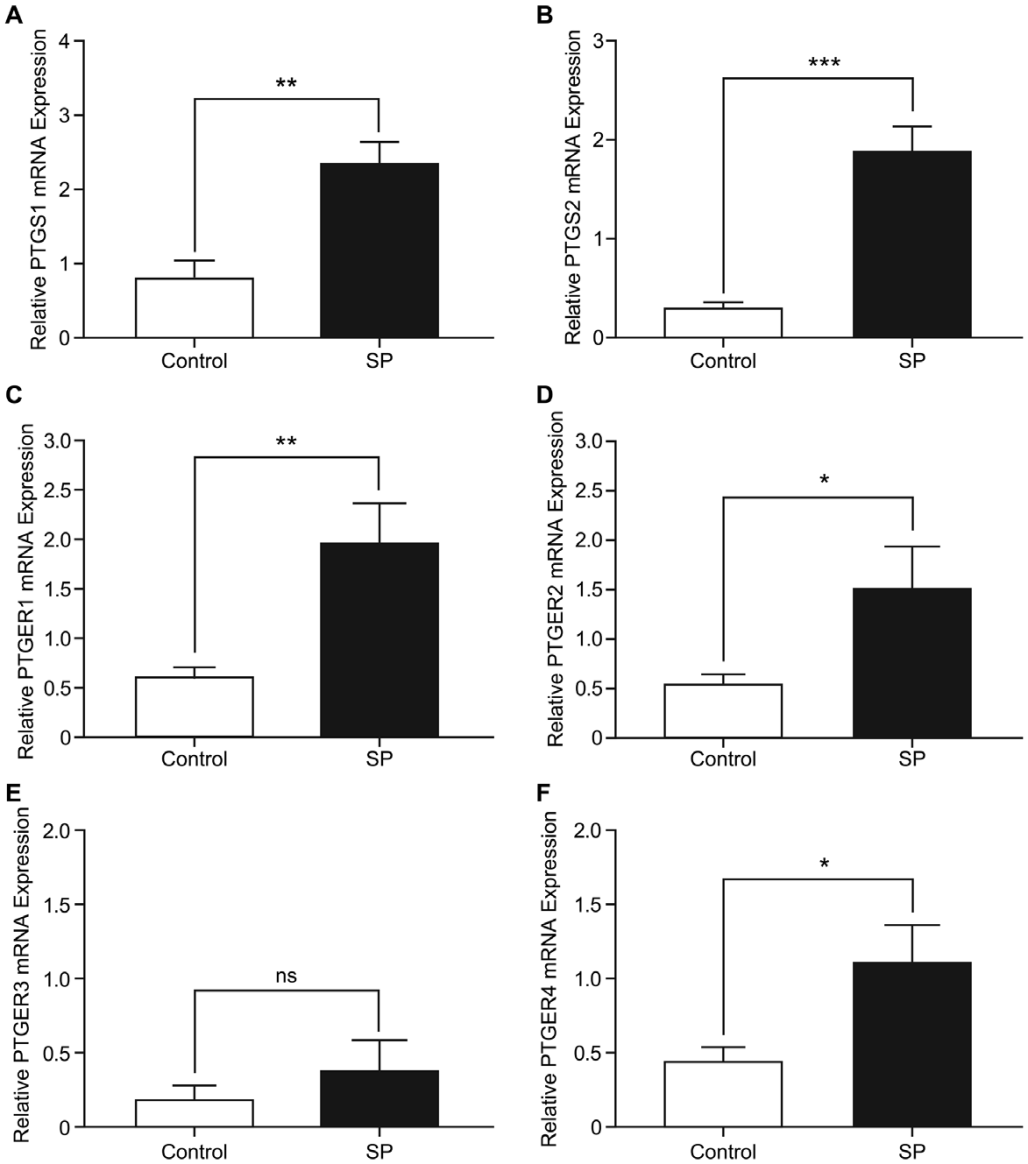
Upregulation of PTGS and PTGER expression in xenograft tumours in response to treatment with seminal plasma. (**A**) PTGS1 and (**B**) PTGS2 mRNA expression was measured by quantitative RT-PCR and is significantly increased by SP (1∶500) as compared to the vehicle (PBS) control, (P<0.01** and P<0.001***) respectively. (**C**) PTGER1 (P<0.01**), (**D**) PTGER2 (P<0.05*), (**E**) PTGER3 (ns) and (**F**) PTGER4 (P<0.05*) mRNA expression levels of the 4 different subtypes of the PGE_2_ receptors as determined by quantitative RT-PCR analysis. Data are represented as mean ± SEM from SP (n = 6) or vehicle controls (n = 8).

We investigated whether expression of the inflammatory PTGS-PTGER pathway was elevated in the xenograft tumours arising from SP-treated animals by quantitative RT-PCR analysis. We found that xenograft tumours from mice treated with SP had significantly elevated expression of PTGS1 (Figure 4A) and PTGS2 (Figure 4B) mRNA compared to controls (3.1±0.1 fold; P<0.01 and 6.2±0.2 fold; P<0.001 for SP vs control for PTGS1 and PTGS2 respectively). Previous in vitro studies from our laboratory have shown that induction of PTGS enzymes coincidently upregulate prostaglandin receptor expression and signalling in vitro [29], [30]. Hence, we investigated the effect of SP treatment on expression of prostaglandin receptors in xenograft tumours. Administration of SP induced the expression of PTGER1 (Figure 4C), PTGER2 (Figure 4D) and PTGER4 (Figure 4F) mRNA in the SP-treated animals compared to the control group (3.2±0.3 fold, P<0.01; 2.9±0.3 fold, P<0.05; 3.1±0.3 fold, P<0.05 for SP vs control for PTGER1, PTGER2 and PTGER4 respectively), but had no significant effect on expression of PTGER3 mRNA (Figure 4E).

### Increased expression of pro-inflammatory mediators IL-6 and IL-11 and pro-angiogenic factor VEGF-A in xenograft tumours in response to treatment with seminal plasma

**Figure 5:**
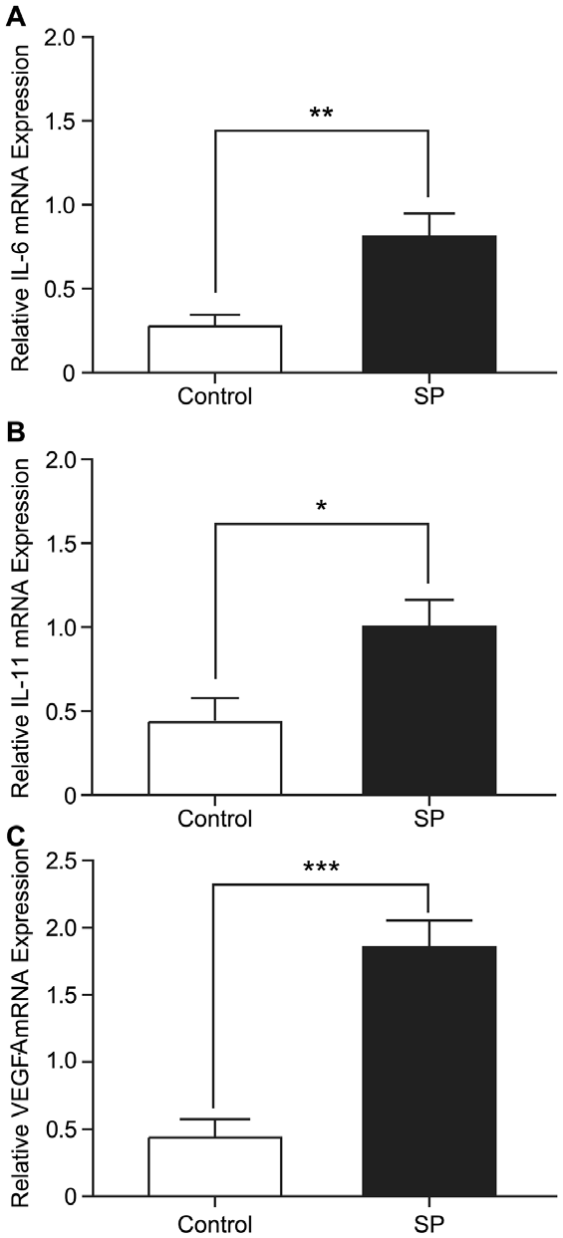
Increased expression of pro-inflammatory cytokines IL-6 and IL-11 and pro-angiogenic factor VEGF-A in xenograft tumours in response to treatment with seminal plasma. (**A**) IL-6 (P<0.01**), (**B**) IL-11 (P<0.05*) and (**C**) VEGF-A (P<0.001***) mRNA expression as determined by quantitative RT-PCR are significantly increased in nude mice xenograft tumours administered with SP (1∶500) in comparison to the vehicle (PBS) control treatment group. Data are represented as mean ± SEM SP (n = 6) or vehicle (PBS) controls (n = 8).

Inflammatory effectors, including growth factors and prostaglandins are known to regulate the tumour microenvironment by inducing pro-inflammatory cytokines and potent proangiogenic factors in order to mobilize immune cells and promote angiogenesis and vascular permeability [8]. We next investigated the expression of potent pro-inflammatory cytokines and angiogenic factors with known roles in tumour development in the tumour xenografts. Quantitative RT-PCR analysis showed a significant increase in expression of IL-6 (Figure 5A) and IL-11 (Figure 5B) mRNA expression in tumour xenografts in SP-treated animals compared to the control group (2.7±0.2 fold, P<0.01 and 2.6±0.1 fold, P<0.05 for SP vs control for IL-6 and IL-11, respectively). Furthermore, we found a significant increase in VEGF-A mRNA expression in xenograft tumours in mice injected with SP in comparison to the control group (4.6±0.2 fold; Figure 5C; P<0.001).

### Blood vessel size is increased in nude mouse xenografts treated with seminal plasma

**Figure 6:**
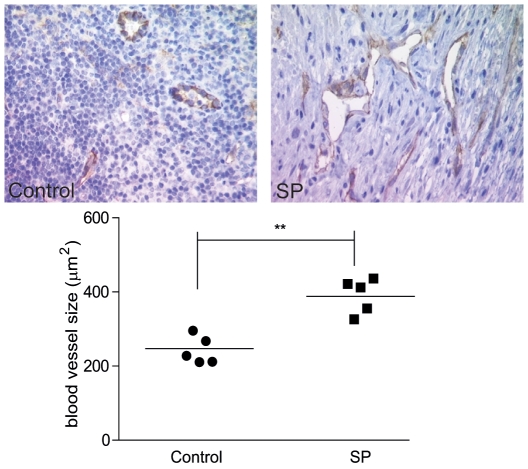
Blood vessel diameter is increased in nude mouse xenografts treated with seminal plasma. Blood vessel size as determined by immunohistochemistry (representative images shown for control and SP-treated animals) and quantitative stereology performed on SP-treated and vehicle control (n = 5 per group) xenograft tumours stained for the endothelial cell marker CD31. The diameter of blood vessels was significantly increased xenograft tumours from animals administered with SP (1∶500) in comparison to the control treatment group (P<0.05*).

Since we identified that the HeLa cell xenograft tumours from mice administered SP had elevated VEGF-A expression, we investigated whether these tumours also displayed a more developed vasculature relative to tumours collected from mice that were treated with vehicle. We performed immunohistochemistry on HeLa cell xenograft tumours for the endothelial cell marker CD31 and quantified blood vessel size by stereology (Figure 6). We found that that SP treatment significantly increased blood vessel size from 247±19 µm^2^ to 388±22 µm^2^ in xenograft tumours (1.7±0.1 fold for SP versus control group respectively; P<0.01). These data suggest that the enhanced tumour growth observed in xenografts arising from mice injected with SP was due to an increase in the capacity of blood vessels to supply the tumour with nutrients and oxygen.

### Seminal plasma induction of IL-6, IL-11 and VEGF-A is mediated via the inflammatory PTGS pathway

**Figure 7:**
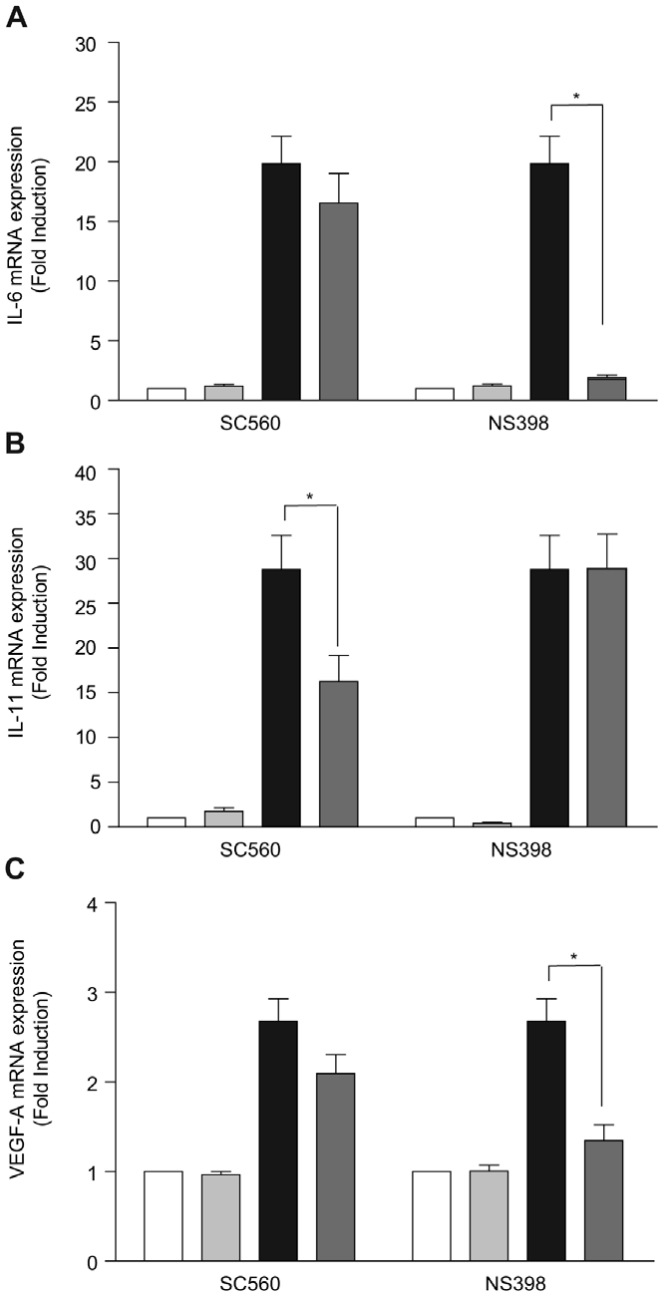
Seminal plasma regulates IL-6, IL-11 and VEGF-A mRNA expression via the inflammatory PTGS pathway . Quantitative RT-PCR analysis of IL-6 (**A**), IL-11 (**B**) and VEGF-A (**C**) expression. HeLa cells were treated for 6 hours with vehicle (white bars), inhibitor alone (light grey bars), SP (1∶100; black bars) or SP and inhibitor (dark grey bars). (P<0.05*) Data are represented as mean ± SEM from 4 independent experiments.

We investigated whether SP-mediated IL-6, IL-11 and VEGF-A expression in vitro is mediated via the induction of the inflammatory PTGS pathway. HeLa cells were treated with 1∶100 dilution of SP (black bar) or vehicle (white bar) for 6 hours in the absence or presence of the selective PTGS1 inhibitor SC560 or selective PTGS2 inhibitor NS398 (dark grey bar) and IL-6, IL-11 and VEGF-A mRNA expression investigated by quantitative real time RT-PCR analysis. We found that co-treatment of HeLa cells with SP and the selective PTGS1 inhibitor SC560 significantly reduced IL-11 (but not IL-6 or VEGF-A) mRNA expression (black bar) compared with vehicle-treated cells (Figure 7B; white bar; P<0.05). By contrast we found that co-treatment of HeLa cells with SP and the selective PTGS2 inhibitor NS398 (dark grey bar; P<0.05) completely abolished SP-mediated IL-6 and VEGF-A mRNA expression (Figure 7A and 7C; black bar) to the levels observed in vehicle treated cells (white bar). However, co-treatment of cells with SP and NS398 had no significant effect on SP-mediated IL-11 expression (Figure 7B). Furthermore, treatment of HeLa cells with SC560 or NS398 alone had negligible effect on basal expression of IL-6, IL-11 or VEGF-A mRNA expression (light grey bar).

### Seminal plasma enhances cellular proliferation and cellular growth in HeLa cells via the PTGS2-PG pathway

**Figure 8:**
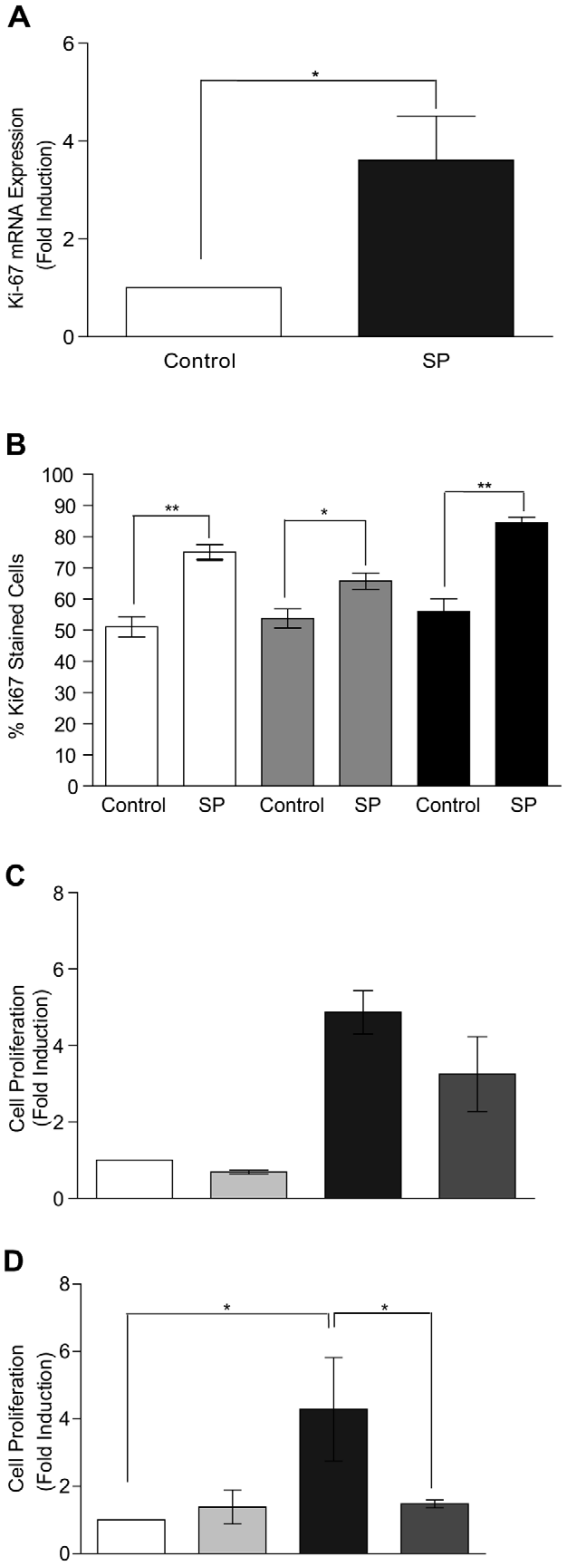
Seminal plasma enhances cellular proliferation and cellular growth in HeLa cells via the PTGS2 pathway. The effects of SP on cell proliferation and cell growth. (**A**) Ki-67 mRNA expression was measured by quantitative RT-PCR analysis and is significantly increased by SP (1∶100) following treatment for 6 hrs in comparison to the vehicle control (P<0.05*). (**B**) Quantitative stereology and immunohistochemistry for Ki-67 on HeLa cells treated with SP (1∶100) for 24, 48 and 72 hrs. The number of positively stained cells were quantified and was shown to be significantly increased in comparison to the vehicle treatment groups, (P<0.05* and P<0.01 respectively). (**C**) Cellular proliferation on HeLa cells treated for 72 hours with vehicle (white bar), SC560 inhibitor alone (light grey bar), SP (1∶100; black bar) or SP and SC560 (dark grey bar). (**D**) Cellular proliferation on HeLa cells treated for 72 hours with vehicle (white bar), NS398 inhibitor alone (light grey bar), SP (1∶100; black bar) or SP and NS398 (dark grey bar). Cellular proliferation was determined as described in the methods (P<0.05*). Data are represented as mean ± SEM from 4 independent experiments.

Finally we investigated a potential mechanism for the SP-induced growth and proliferation of neoplastic cervical epithelial cells in vitro. HeLa cells were treated with SP (1∶100) or vehicle for 6 hours and the RNA subjected to quantitative real time PCR for the proliferation marker Ki-67. We found that SP significantly increased the expression of Ki-67 mRNA when compared to the vehicle control (Figure 8A; 3.6±0.9 fold, P<0.05). We confirmed the increase in Ki-67 expression in SP-treated HeLa cells by immunohistochemistry and quantitative stereology. Cells were treated with SP (1∶100) or vehicle for 24, 48 or 72 hours. We found a significant increase in Ki-67 immunoreactivity at all time points investigated in response to SP (1∶100) stimulation (Figure 8B). In order to determine a mechanism for the enhanced xenograft tumour growth rate in vivo, we investigated whether SP-mediated HeLa cell proliferation was mediated via the inflammatory PTGS pathway. HeLa cells were treated with 1∶100 dilution of SP (black bar) or vehicle (white bar) for 72 hours in the absence or presence of the selective PTGS1 inhibitor SC560 or selective PTGS2 inhibitor NS398 (dark grey bar, Figure 8C and 8D, respectively). We found that although co-treatment of HeLa cells with SP and the selective PTGS1 inhibitor marginally reduced cellular proliferation (Figure 8C, dark grey bar), the reduction was not statistically significant. However, co-treatment of cells with SP and the selective PTGS2 inhibitor NS398 completely abolished the SP-mediated increase in cellular proliferation (Figure 8D, dark grey bar; P<0.05). Incubation of HeLa cells with inhibitor alone (Figure 8C and 8D, light grey bar) had no significant effect on basal cell proliferation.

## Discussion

A functional relationship between inflammation and cancer has been long established with Rudolf Virchow hypothesizing as early as 1863 that the beginnings of cancer originated from chronic sites of inflammation [8]. Cancer of the cervix remains the most prolific female cancer in developing countries with a notable decline in mortality rates in Western countries being ascribed to successful screening programmes and vaccine development [31], [32]. Although HPV infection of the cervical epithelium is well regarded as the main cause of cervical cancer, it takes on average 10–15 years for the development of invasive disease. These observations have led to the hypothesis that alternative biological cues and causative factors in addition to persistent HPV infection are required for the transition of precursor lesions to cervical cancer [33], [34]. Although intercourse with multiple sexual partners has been implicated in the etiology of cervical cancer, the impact of seminal fluid on the neoplastic cervical epithelium and its role in regulating disease progression has yet to be fully investigated.

It has recently been shown that seminal fluid induces an inflammatory response in the cervix in humans after coitus, characterised by the influx of leukocytes and dendritic cells into the epithelium and stromal compartments and an accompanying increase in inflammatory cytokines such as IL-6 and IL-8 [24]. In the present study we show that direct exposure of neoplastic cervical epithelial cells to SP in vitro can increase the expression of both PTGS1 and PTGS2. We and others have previously shown this pro-inflammatory PTGS-prostaglandin pathway to be up-regulated in cervical cancer [10], [11], [12], [13]. Elevated biosynthesis of prostaglandins as a consequence of elevated PTGS enzyme expression is a critical step in initiating inflammation and the tissue remodeling associated with inflammation and cancer [7]. PG biosynthesis and signaling has been shown to enhance tumorigenesis in human cancers [35], [36]. The induction of this pathway directly by SP suggests that in sexually active women, repeated exposure of neoplastic cervical epithelial cells to SP can potentially exacerbate the inflammatory actions of this pathway and enhance disease progression.

Inflammatory effectors, including growth factors and prostaglandins are known to regulate the tumour microenvironment by inducing pro-inflammatory cytokines/chemokines to modulate neutrophil infiltration into tumours [37] and potentially alter vascular permeability [38], [39]. In our study, we show that in addition to the PTGS-PG pathway, direct SP stimulation of HeLa cells can enhance the expression of pro-inflammatory cytokines IL-6 and IL-11 and the pro-angiogenic factor VEGF-A. These mediators are involved in many regulatory roles but their increased expression has been associated with several reproductive tract cancers, including endometrial cancer, ovarian cell carcinoma and squamous cell carcinoma of the uterine cervix [40], [41], [42], [43], [44], [45]. The induced expression of these effector molecules in neoplastic cervical epithelial cells by direct stimulation with SP suggests that repeated exposure of the cervix to seminal fluid in the absence of barrier contraception could exacerbate tumour-associated inflammation and could enhance disease progression.

In addition to direct exposure of the epithelial layer surrounding the anterior and posterior vaginal fornix, ecto- and endocervix to seminal fluid during intercourse, direct absorption of components of SP can enter into the endometrial or peritoneal bed. The intravaginal absorption of components of SP have been rigorously debated [46], however vaginal absorption of molecules is widely regarded as a potential means of drug delivery in women. Absorption may occur as a result of hematogenous dissemination or direct tissue perfusion through the anterior or posterior vaginal fornix [27]. Although it is likely that the concentrations of inflammatory mediators in SP in the recipient woman after intercourse would be orders of magnitude lower than that found in semen, concentrations of inflammatory mediators would nevertheless be greater in sexually active women and could impact in situ on tumour growth.

In order to test whether raised systemic levels of inflammatory mediators present in SP could enhance the growth rate of neoplastic cervical epithelial cells and hence enhance tumour progression, we used a nude mouse model system. We engrafted HeLa cells subcutaneously into the dorsal flank of nude mice. Following engraftment, animals were divided randomly into two groups and administered seminal plasma or vehicle control via intra-peritoneal injection. We used a five-fold lower concentration of seminal plasma for the in vivo study and used an intra-peritoneal route of administration to simulate the lower levels of inflammatory mediators which would impact on neoplastic cervical cell function in vivo, following diffusion into the endometrial or peritoneal bed, compared with direct stimulation of cells present in the endocervix at intercourse as discussed earlier in our study. We found that HeLa cell xenografts in mice administered SP grew at an accelerated rate resulting in tumours of approximately twice the size and weight compared with controls. These observations are similar to those of Liu and colleagues, who recently showed that HeLa cells pre-treated with seminal plasma and engrafted into nude mice grew at an accelerated rate compared with control cells [47]. We investigated whether the increase in tumour growth was due to cellular proliferation of the engrafted HeLa cells. Quantitative real time RT-PCR analysis and immunohistochemistry for the proliferation marker Ki67 confirmed that the increased size, weight and growth rate for xenografts from SP treated animals was due to accelerated rate of HeLa cell proliferation. Coincident with the enhanced tumour growth rate and size, we found an increase in expression of components of the PTGS-PG pathway. As observed for our in vitro studies, we found that SP enhanced the expression of the inflammatory PTGS1 and PTGS2 enzymes and also elevated expression of the E-series prostaglandin receptors PTGER1, PTGER2 and PTGER4. We have previously shown that PTGS1, PTGS2, PTGER2 and PTGER4 are elevated in cervical cancers [11].

Our present study also shows that intra-peritoneal administration of SP can dramatically elevate the expression of pro-inflammatory cytokines IL-6 and IL-11 in HeLa cell xenograft tumours. Although the molecular mechanism whereby SP regulates these cytokines is unclear, recent studies confirm that SP can enhance expression of a host of cytokines, including IL-6, IL-10 and monocyte chemoattractant protein-1 (MCP-1) in cervical and vaginal epithelial cells vitro [48], [49], [50]. Expression and production of inflammatory cytokines, including IL-6, are elevated in carcinoma of the cervix, where they act as autocrine growth factors to regulate the inflammatory response via induction of the PTGS2-PGE_2_ signal transduction pathway [40], [51]. This is further supported by studies in mammals that show that seminal plasma possesses the ability to interact with cervical and uterine epithelial cells and trigger the release of pro-inflammatory cytokines such as IL-6 which in turn induces local cellular and molecular changes and immune cell recruitment which can promote an inflammatory response [24], [50], [52]. Therefore, SP can be deemed as a regulator of pro-inflammatory cytokines that in turn contributes to controlling immune cell function and inflammation, which can promote tumour progression.

In order for sustained tumour growth, an enhanced blood supply is needed for the provision of necessary nutrients and oxygen. Similar to our observation in vitro, we have shown that seminal plasma significantly induced expression of the potent angiogenic factor VEGF-A in xenograft tumours in vivo and enhances blood vessel size. We believe that the larger vessel size would permit greater delivery of oxygen and nutrients into the tumour and facilitate more rapid growth thus leading to larger xenograft tumours in SP treated animals in comparison to the control treatment group. Indeed several studies have previously shown that VEGF-A influences vessel size and therefore has an impact on the angiogenesis in cervical cancer [53], [54]. CD31 expression, as a marker of angiogenesis has been correlated with microvessel density and metastases in numerous solid tumours, including carcinomas of breast, lung and prostate [55], [56], [57], [58]. A recent study performed by Mazibrada and colleagues showed that sections of invasive cervical cancer have significantly more CD31 expression in comparison to normal tissues [59]. The mechanisms whereby SP enhances blood vessel size in vivo is unclear, however we have shown in vitro that VEGF-A expression is regulated by the PG pathway. We have previously demonstrated that PGE_2_ and seminal plasma (1∶500 dilution) can regulate VEGF-A expression in HeLa cells in vitro via PTGER4-mediated activation of the epidermal growth factor receptor and extracellular signal-regulated kinase pathways [16]. It is therefore likely that the increased expression of VEGF-A induced by SP in HeLa cell xenografts could alter vascular function and that this could likely be mediated by PGE_2_ present in the SP via the elevated PTGER receptors in the SP-treated xenografts. Furthermore, our data using selective inhibition of PTGS enzyme activity confirm that PTGS-PG pathway is central to regulating SP-mediated inflammatory and angiogenic gene expression. These results are consistent with our previous finding using a HeLa TeToff system for inducible expression of PTGS1, where we demonstrated the molecular mechanism for regulation of prostaglandin receptors and angiogenic factors in HeLa cells by PTGS1, via the induction of PTGS2 and biosynthesis of PGE_2_ [12]. Although infiltrating immune cells into tumours have been associated with angiogenesis [60], we did not observe any differences in immune cell infiltrate between the control or SP-treated animals, so it is unlikely that the alterations in vascular size we observed in the present study are due to immune cell infiltration.

To investigate whether SP directly impacted on cellular proliferation, we treated HeLa cells with SP in vitro. We found that SP significantly enhanced the expression of the proliferation marker Ki67 in HeLa cells and increased cellular proliferation. Furthermore we found that the SP-mediated increase in HeLa cell proliferation was mediated by PTGS2, since treatment of cells with the PTGS2 inhibitor NS398, but not the selective PTGS1 inhibitor SC560, abolished the SP-mediated increase in cellular proliferation.

Seminal plasma is known to contain many inflammatory agents such as prostaglandins, transforming growth factor-ß (TGF-ß), and glycoprotein signalling molecules including cytokines and growth factors [21]. These molecules are independently thought to induce cellular and molecular changes in the cervical epithelium by binding to specific target cells in the female reproductive tract, thus modulating gene expression, cellular composition and structure and function of local tissue via cytokine synthesis as well as by release of other autocrine and paracrine factors [61]. In our study we have not attempted to isolate the precise signaling molecules in the SP responsible for orchestrating the effects we observed in vitro and in vivo. It is likely that the net effect we observe in relation to cervical cancer cell growth and inflammation is a result of the integration of all signalling inputs into HeLa cells in response to SP.

### Conclusions

Our study has demonstrated that SP can mediate an inflammatory response by upregulating the expression of pro-inflammatory enzymes PTGS1 and PTGS2, pro-inflammatory cytokines IL-6 and IL-11 and the proangiogenic factor VEGF-A in vitro and in nude mice in vivo. Furthermore we have demonstrated that a raised systemic inflammatory response simulated by intra-peritoneal administration of SP can enhance the growth rate and size of HeLa cell xenograft tumours in nude mice. Coincident with the enhanced growth rate in SP-treated xenograft tumours, we found a significant alteration in the size of the blood vessels supporting the SP-treated xenograft tumours. These findings indicate gross tissue remodelling events in these tumours in response to SP to facilitate greater nutrient supply to support the enhanced growth rate. Finally we show in vitro, that SP induces IL-6, IL-11 and VEGF-A expression and promotes HeLa cell proliferation via induction of the inflammatory PTGS pathway. Taken together our data strongly support a role for SP in enhancing cervical tumorigenesis in sexually active women via the induction of inflammatory pathways.

## References

[1] Arbyn M, Castellsague X, de Sanjose S, Bruni L, Saraiya M (2011). Worldwide burden of cervical cancer in 2008.. Ann Oncol.

[2] Anorlu RI (2008). Cervical cancer: the sub-Saharan African perspective.. Reprod Health Matters.

[3] Canadas MP, Videla S, Darwich L, Tarrats A, Pinol M (2010). Human papillomavirus HPV-16, 18, 52 and 58 integration in cervical cells of HIV-1-infected women.. J Clin Virol.

[4] Al-Daraji WI, Smith JH (2009). Infection and cervical neoplasia: facts and fiction.. Int J Clin Exp Pathol.

[5] Boccardo E, Lepique AP, Villa LL (2010). The role of inflammation in HPV carcinogenesis.. Carcinogenesis.

[6] Subbaramaiah K, Dannenberg AJ (2007). Cyclooxygenase-2 transcription is regulated by human papillomavirus 16 E6 and E7 oncoproteins: evidence of a corepressor/coactivator exchange.. Cancer Res.

[7] Jabbour HN, Sales KJ, Catalano RD, Norman JE (2009). Inflammatory pathways in female reproductive health and disease.. Reproduction.

[8] Coussens LM, Werb Z (2002). Inflammation and cancer.. Nature.

[9] Goswami B, Rajappa M, Sharma M, Sharma A (2008). Inflammation: its role and interplay in the development of cancer, with special focus on gynecological malignancies.. Int J Gynecol Cancer.

[10] Ryu HS, Chang KH, Yang HW, Kim MS, Kwon HC (2000). High cyclooxygenase-2 expression in stage IB cervical cancer with lymph node metastasis or parametrial invasion.. Gynecol Oncol.

[11] Sales KJ, Katz AA, Davis M, Hinz S, Soeters RP (2001). Cyclooxygenase-2 expression and prostaglandin E2 synthesis are up- regulated in carcinomas of the cervix: a possible autocrine/paracrine regulation of neoplastic cell function via EP2/EP4 receptors.. J Clin Endocrinol Metab.

[12] Sales KJ, Katz AA, Howard B, Soeters RP, Millar RP (2002). Cyclooxygenase-1 is up-regulated in cervical carcinomas: autocrine/paracrine regulation of cyclooxygenase-2, prostaglandin e receptors, and angiogenic factors by cyclooxygenase-1.. Cancer Res.

[13] Oh JM, Kim SH, Lee YI, Seo M, Kim SY (2009). Human papillomavirus E5 protein induces expression of the EP4 subtype of prostaglandin E2 receptor in cyclic AMP response element-dependent pathways in cervical cancer cells.. Carcinogenesis.

[14] Smith WL, DeWitt DL, Garavito RM (2000). CYCLOOXYGENASES: structural, cellular, and molecular biology.. Annu Rev Biochem.

[15] Battersby S, Sales KJ, Williams AR, Anderson RA, Gardner S (2007). Seminal plasma and prostaglandin E2 up-regulate fibroblast growth factor 2 expression in endometrial adenocarcinoma cells via E-series prostanoid-2 receptor-mediated transactivation of the epidermal growth factor receptor and extracellular signal-regulated kinase pathway.. Hum Reprod.

[16] Muller M, Sales KJ, Katz AA, Jabbour HN (2006). Seminal plasma promotes the expression of tumorigenic and angiogenic genes in cervical adenocarcinoma cells via the E-series prostanoid 4 receptor.. Endocrinology.

[17] Sales KJ, Katz AA, Millar RP, Jabbour HN (2002). Seminal plasma activates cyclooxygenase-2 and prostaglandin E2 receptor expression and signalling in cervical adenocarcinoma cells.. Mol Hum Reprod.

[18] Ness RB, Grainger DA (2008). Male reproductive proteins and reproductive outcomes.. Am J Obstet Gynecol.

[19] Fung KY, Glode LM, Green S, Duncan MW (2004). A comprehensive characterization of the peptide and protein constituents of human seminal fluid.. Prostate.

[20] Robertson SA, Guerin LR, Moldenhauer LM, Hayball JD (2009). Activating T regulatory cells for tolerance in early pregnancy - the contribution of seminal fluid.. J Reprod Immunol.

[21] Robertson SA (2005). Seminal plasma and male factor signalling in the female reproductive tract.. Cell Tissue Res.

[22] Robertson SA, Guerin LR, Bromfield JJ, Branson KM, Ahlstrom AC (2009). Seminal fluid drives expansion of the CD4+CD25+ T regulatory cell pool and induces tolerance to paternal alloantigens in mice.. Biol Reprod.

[23] Moldenhauer LM, Diener KR, Thring DM, Brown MP, Hayball JD (2009). Cross-presentation of male seminal fluid antigens elicits T cell activation to initiate the female immune response to pregnancy.. J Immunol.

[24] Sharkey DJ, Tremellen KP, Jasper MJ, Gemzell-Danielsson, Robertson SA (2012). Seminal Fluid induces leukocyte recruitment and cytokine and chemokine mRNA expression in the human cervix after coitus.. J Immunol.

[25] Jeremias J, David SS, Toth M, Witkin SS (1997). Induction of messenger RNA for the 70 kDa heat shock protein in HeLa cells and the human endocervix following exposure to semen: Implications for antisperm antibody production and susceptibility to sexually transmitted infections.. Hum Reprod.

[26] Jeremias J, Witkin SS (1999). Effect of human seminal fluid on production of messenger ribonucleic acid for metalloproteinase 2 and metalloproteinase 9 in cervical epithelial carcinoma cells.. Am J Obstet Gynecol.

[27] Klemmt L, Scialli AR (2005). The transport of chemicals in semen.. Birth Defects Res B Dev Reprod Toxicol.

[28] Catalano RD, Wilson MR, Boddy SC, McKinlay AT, Sales KJ (2011). Hypoxia and prostaglandin e receptor 4 signalling pathways synergise to promote endometrial adenocarcinoma cell proliferation and tumour growth.. PLoS One.

[29] Sales KJ, Jabbour HN (2003). Cyclooxygenase enzymes and prostaglandins in pathology of the endometrium.. Reproduction.

[30] Jabbour HN, Sales KJ (2004). Prostaglandin receptor signalling and function in human endometrial pathology.. Trends Endocrinol Metab.

[31] Ponten J, Adami HO, Bergstrom R, Dillner J, Friberg LG (1995). Strategies for global control of cervical cancer.. Int J Cancer.

[32] Harlan LC, Bernstein AB, Kessler LG (1991). Cervical cancer screening: who is not screened and why?. Am J Public Health.

[33] Chan JK, Monk BJ, Brewer C, Keefe KA, Osann K (2003). HPV infection and number of lifetime sexual partners are strong predictors for ‘natural’ regression of CIN 2 and 3.. Br J Cancer.

[34] Schiffman MH, Brinton LA (1995). The epidemiology of cervical carcinogenesis.. Cancer.

[35] Sales KJ, Jabbour HN (2003). Cyclooxygenase enzymes and prostaglandins in reproductive tract physiology and pathology.. Prostaglandins Other Lipid Mediat.

[36] Rizzo MT (2011). Cyclooxygenase-2 in oncogenesis.. Clin Chim Acta.

[37] Wallace AE, Sales KJ, Catalano RD, Anderson RA, Williams AR (2009). Prostaglandin F2alpha-F-Prostanoid Receptor Signaling Promotes Neutrophil Chemotaxis via Chemokine (C-X-C Motif) Ligand 1 in Endometrial Adenocarcinoma.. Cancer Res.

[38] Takehara H, Iwamoto J, Mizokami Y, Takahashi K, Ootubo T (2006). Involvement of cyclooxygenase-2-prostaglandin E2 pathway in interleukin-8 production in gastric cancer cells.. Dig Dis Sci.

[39] Toomey DP, Murphy JF, Conlon KC (2009). COX-2, VEGF and tumour angiogenesis.. Surgeon.

[40] Eustace D, Han X, Gooding R, Rowbottom A, Riches P (1993). Interleukin-6 (IL-6) functions as an autocrine growth factor in cervical carcinomas in vitro.. Gynecol Oncol.

[41] Fujimoto J, Sakaguchi H, Aoki I, Tamaya T (2000). Clinical implications of expression of interleukin 8 related to angiogenesis in uterine cervical cancers.. Cancer Res.

[42] Venkatakrishnan G, Salgia R, Groopman JE (2000). Chemokine receptors CXCR-1/2 activate mitogen-activated protein kinase via the epidermal growth factor receptor in ovarian cancer cells.. J Biol Chem.

[43] Watson JM, Sensintaffar JL, Berek JS, Martinez-Maza O (1990). Constitutive production of interleukin 6 by ovarian cancer cell lines and by primary ovarian tumor cultures.. Cancer Res.

[44] Sales KJ, Grant V, Cook IH, Maldonado-Perez D, Anderson RA (2010). Interleukin-11 in endometrial adenocarcinoma is regulated by prostaglandin F2alpha-F-prostanoid receptor interaction via the calcium-calcineurin-nuclear factor of activated T cells pathway and negatively regulated by the regulator of calcineurin-1.. Am J Pathol.

[45] Sales KJ, Maldonado-Perez D, Grant V, Catalano RD, Wilson MR (2009). Prostaglandin F2alpha-F-prostanoid receptor regulates CXCL8 expression in endometrial adenocarcinoma cells via the calcium-calcineurin-NFAT pathway.. Biochim Biophys Acta.

[46] Ney PG (1986). The intravaginal absorption of male generated hormones and their possible effect on female behaviour.. Med Hypotheses.

[47] Liu L, Liu C, Lou F, Zhang G, Wang X (2011). Activation of telomerase by seminal plasma in malignant and normal cervical epithelial cells.. J Pathol.

[48] Sharkey DJ, Macpherson AM, Tremellen KP, Robertson SA (2007). Seminal plasma differentially regulates inflammatory cytokine gene expression in human cervical and vaginal epithelial cells.. Mol Hum Reprod.

[49] Denison FC, Grant VE, Calder AA, Kelly RW (1999). Seminal plasma components stimulate interleukin-8 and interleukin-10 release.. Mol Hum Reprod.

[50] Robertson SA, Mau VJ, Tremellen KP, Seamark RF (1996). Role of high molecular weight seminal vesicle proteins in eliciting the uterine inflammatory response to semen in mice.. J Reprod Fertil.

[51] Takano H, Harigaya K, Ishii G, Sugaya Y, Soeta S (1996). Interleukin-6 (IL-6) production in carcinoma of the cervix.. Arch Gynecol Obstet.

[52] Robertson SA, Mayrhofer G, Seamark RF (1992). Uterine epithelial cells synthesize granulocyte-macrophage colony- stimulating factor and interleukin-6 in pregnant and nonpregnant mice.. Biol Reprod.

[53] Guidi AJ, Abu-Jawdeh G, Berse B, Jackman RW, Tognazzi K (1995). Vascular permeability factor (vascular endothelial growth factor) expression and angiogenesis in cervical neoplasia.. J Natl Cancer Inst.

[54] Cheng WF, Chen CA, Lee CN, Chen TM, Hsieh FJ (1999). Vascular endothelial growth factor in cervical carcinoma.. Obstet Gynecol.

[55] Darai E, Bringuier AF, Walker-Combrouze F, Fauconnier A, Couvelard A (1998). CD31 expression in benign, borderline, and malignant epithelial ovarian tumors: an immunohistochemical and serological analysis.. Gynecol Oncol.

[56] Horak ER, Leek R, Klenk N, LeJeune S, Smith K (1992). Angiogenesis, assessed by platelet/endothelial cell adhesion molecule antibodies, as indicator of node metastases and survival in breast cancer.. Lancet.

[57] Yamazaki K, Abe S, Takekawa H, Sukoh N, Watanabe N (1994). Tumor angiogenesis in human lung adenocarcinoma.. Cancer.

[58] Fregene TA, Khanuja PS, Noto AC, Gehani SK, Van Egmont EM (1993). Tumor-associated angiogenesis in prostate cancer.. Anticancer Res.

[59] Mazibrada J, Ritta M, Mondini M, De Andrea M, Azzimonti B (2008). Interaction between inflammation and angiogenesis during different stages of cervical carcinogenesis.. Gynecol Oncol.

[60] Quigley JP, Deryugina EI (2012). Combating angiogenesis early: potential of targeting tumor-recruited neutrophils in cancer therapy.. Future Oncol.

[61] Aumuller G, Riva A (1992). Morphology and functions of the human seminal vesicle.. Andrologia.

